# Dietary quality indices modify the effects of apolipoprotein B polymorphisms on biochemical and anthropometric factors in type 2 diabetes mellitus

**DOI:** 10.1038/s41598-021-01884-1

**Published:** 2021-11-17

**Authors:** Elmira Karimi, Gity Sotoudeh, Masoumeh Rafiee, Fariba Koohdani

**Affiliations:** 1 Department of Community Nutrition, School of Nutritional Sciences and Dietetics, Tehran University of Medical Sciences, Tehran, Iran; 2 Department of Clinical Nutrition, School of Nutrition and Food Science, Isfahan University of Medical Sciences, Isfahan, Iran; 3 Diabetes Research Center, Endocrinology and Metabolism Clinical Sciences Institute, Tehran University of Medical Sciences, Tehran, Iran; 4 Department of Cellular and Molecular Nutrition, School of Nutritional Sciences and Dietetics, Tehran University of Medical Sciences, PO Box 141556117, Tehran, Iran

**Keywords:** Predictive markers, Genetics, Biomarkers

## Abstract

We tried to identify the interaction between dietary quality indices and apolipoprotein B *Ins/Del* and *EcoR1* polymorphisms on biochemical and anthropometric factors in patients with type 2 diabetes mellitus (T2DM). This cross-sectional study recruited 700 adults with T2DM in Tehran. The genotypes of *Ins/Del* and *EcoR1* single nucleotide polymorphisms (SNP) were explored via polymerase chain reaction (PCR). Dietary quality index-international (DQI-I), healthy eating index-2015 (HEI-2015) and dietary phytochemical index (DPI) were calculated by semi-quantitative food frequency questionnaire (FFQ). In both crude and adjusted model for confounding factors, we observed significant interactions between DQI-I and *Ins/Del* SNP on leptin in and 8-iso-prostaglandin F2 α (8-iso-PGF2α), DPI and *EcoR1* SNP on total cholesterol (TC) and between *Ins/Del* SNP and HEI-2015 on interleukin-18 (IL-18). Furthermore, in crude model there were close to meaningful interactions between *EcoR1* SNP and DQI-I on total antioxidant capacity (TAC) and between *EcoR1* SNP and HEI-2015 on serum leptin and superoxide dismutase (SOD) levels. Our finding indicated that the association between DQI-I, HEI-2015 and DPI with IL-18, TC, leptin and 8-iso-PGF2α in patients with T2DM might be dependent on *Ins/Del* and *EcoR1* variants in ApoB gene.

## Introduction

Type 2 diabetes mellitus (T2DM) is a pandemic disease associated to insulin resistance, beta-cell dysfunction and hyperglycemia^1^. Epidemiological studies reported that worldwide prevalence of diabetes mellitus (DM) was about 451 million in 2017 with most being T2DM^2^. Majority of T2DM patients present with biochemical abnormalities including elevated oxidative stress, inflammatory status, disturbed lipid profile^3–6^. High appetite and obesity is another common symptom in T2DM patients that is characterized by high BMI, high blood ghrelin and low blood leptin levels^7^.

There are important relationships between many of these abnormalities and further progression of T2DM disease and other adverse chronic conditions^8^. The causes of hyperglycemia, hyperglycemic-induced oxidative stress and inflammation and dyslipidemia and then T2DM development are multifactorial, and efforts to identify these causes are necessary for the development of beneficial preventive strategies. In addition to modifiable factors such as physical activity and dietary intake, T2DM is affected by non-modifiable factors such as genetic predisposition^9^. Furthermore, recent studies have indicated that genetic variation interact with potential environmental factors such as dietary patterns and intake of food components and hence influence development of T2DM^10,11^. The assessment of gene-diet interaction is called nutrigenetics.

The aim of nutrigenetics is understanding the concept of “personalized nutrition”, which targets to inhibit the progression of chronic diseases by creating dietary recommendations based on individual’s genetic predisposition^12^. Until now, genome-wide investigations have revealed the biological roles of several single-nucleotide polymorphisms (SNPs) in the pathogens of DM^13^.

Since Apo-lipoprotein B (Apo B) participates in the cellular cholesterol uptake from cholesterol-rich lipoproteins^14^, single-nucleotide polymorphism (SNP) in Apo B gene could predict changes in hypercholesterolemia and subsequent oxidative, inflammatory, lipid and anthropometrics states^15,16^. There has been high evidence by far for the Apo B ECOR1 and Ins/Del variants in the Apo B gene, with the E- and Del alleles being probably linked to increasing T2DM risk factors^17,18^. These SNPs have also been indicated to modify associations to dietary components intakes for T2DM -related phenotypes. For instance it has been demonstrated that, in people carrying Del allele in Apo B Ins/Del SNP, there is a higher probability of elevated blood low density lipoprotein (LDL), total cholesterol (TC) and waist to hip ratio (WHR) compared to subjects with Ins/Ins genotype. However, it was later found that low calorie diet and omega-3 poly-unsaturated fatty acids (W-3 PUFA) intake can significantly reduce LDL concentrations and waist circumference in Del allele carriers^19–22^. Moreover, homozygous E + E + genotypes have been observed to have lower probability of elevated BMI in response to a high calorie and saturated fat diet, compared to those with E − allele^23^. However, there have been significantly low nutrigenetics studies on the interaction between dietary patterns and ECOR1 and Ins/Del SNPs with regard to biochemical and anthropometric markers.

Evaluating dietary pattern by various validated indices is an effective way to demonstrate a people’s dietary status. Because, a single food group may not make significant effect on various aspects of health^24^. Dietary quality index-international (DQI-I) and healthy eating index (HEI-2015) are among popular dietary indicators developed to assess overall quality of the diet in different populations. Dietary phytochemical index (DPI) measures total dietary phytochemical content and ranks persons according to their intakes of phytochemical rich foods^25^. A number of studies have also suggested that adherence to healthy diet presented by high scores of DQI-I, HEI-2015 and DPI is negatively associated with lower level of T2DM risk factors^26,27^.

It is unclear if interactions of Apo B ECOR1 and Ins/Del SNPs with dietary intake affect cardiovascular risk factors levels in different populations. In this study, using Iranian subjects with T2DM, we aimed to evaluate if scores of DQI-I, HEI-2015 and DPI modifies associations of biochemical and anthropometric factors and Apo B ECOR1 and Ins/Del SNPs.

## Methods

### Study design and subjects

We conducted an observational cross-sectional study on patients with T2DM. Sample size calculating was through following formula:

N = (([(Z1 − α + Z1 − β) × √1 − r^2^]/r)^2^ + 2), When r = 0.15 β = 0.95 and α = 0.05, the N was equal to 694. Due to data availability, we recruited 700 participants. Subjects were recruited by multistage cluster random sampling method from individuals who visited diabetes referral centers in different regions of Tehran, Iran during June 2011 to October 2012. Subjects were eligible for participation if they were adult and had been previously diagnosed with T2DM based on the decision of the endocrinologist. We excluded pregnant and lactating women and patients who had a current or a previous 2 months history of malignancies, abnormalities in kidney, hepatic, thyroid and cardiovascular system, alcohol intake, dependency on cigarette or drug, consumption of anti-inflammatory medications or dietary supplements. Additionally, subjects with an ongoing insulin therapy were not allowed to enter this research. There was no source of bias in present research. All of the methods of this research were carried out in accordance with the Declaration of Helsinki^28^. All stages of this study had been approved by the Ethics Committee of Tehran University of Medical Sciences (Ethics number: IR.TUMS.VCR.REC.1395.15060). In addition, all eligible individuals signed a declaration on consent to participate after they were informed about aims and protocol of the study.

### Assessment of sociodemographic, anthropometric and physical activity variables

Eligible participants were answered the questionnaires regarding their sociodemographic information such as age, job, medical history, duration and family history of T2DM, smoking and alcohol usage and consumption of lipid lowering medications. Assessment of physical activity was done using a validated questionnaire that was based on metabolic equivalent to task (MET-h/day) of daily activities^29^. Weight (kg) and height (m) were measured via Seca falcon scales (Seca, Germany) by standard protocols^30^. Waist circumference was measured at the level midway between the lower margin of the last palpable rib and the top of the iliac crest at the end of several consecutive natural breaths. BMI was calculated via the following formula: weight/height^2^ (kg/m^2^).

### Assessment of healthy eating index (HEI-2015), dietary quality index international (DQI-I) and dietary phytochemical index (DPI)

The participant’s usual dietary intake on a daily, weekly, monthly or annual basis during last year was assessed through face-to-face interviews performed by trained nutritionists using a validated semi-quantitative food frequency questionnaire (FFQ) for Iranian population^31^. The detailed structures of HEI-2015, DQI-I and DPI have been numerously described elsewhere^32–34^. Healthy eating index-2015 (HEI-2015) is a new tool for prediction of the adherence level to 2015–2020 DGA. In brief, for total HEI-2015 score calculation, FFQ items were classified in to 13 food groups including (1) Total Vegetables, (2) Dairy, (3) Whole Fruits, (4) Total Fruits, (5) Total Protein Foods, (6) Greens and Beans, (7) Seafood and Plant Proteins, (8) Whole Grains, (9) Refined Grains, (10) Added Sugars, (11) Saturated Fats, (12) Fatty Acids and (13) Sodium. In this classification, there were similar score ranges (0–5) for all parameters except for number 1–6 food groups which had a score range between 1 and 10. Scores of 13 food parameters were added to obtain the final HEI-2015 score in which 0 and 100 were indicative of minimum and maximum adherence to 2015–2020 DGA respectively. In DQI-I score, variety component (total score: 0–20) composed of overall diversity between food groups (scores: 0–15) and diversity within protein food sources (scores: 0–5). In adequacy component (total score: 0–40), there were scores ranging from 0 to 5 for investigation of sufficient intakes of 8 parameters including fruits, grains, fiber, protein, iron, calcium and vitamin C. Moderation component (total score: 0–30) investigated restraint intake of 5 parameters (each of them had scores ranging from 0 to 6) including total fat, saturated fat, sodium, cholesterol and empty calorie food. Overall balance (total score: 0–10) was also according to the proper ratio of macronutrient intake including fat, carbohydrate and protein (score 0–6) and fatty acids intake including saturated fatty acids (SFA), PUFA and monounsaturated fatty acids (MUFA) (score 0–4). Scores of four aspects were summed up to obtain the total score of DQI-I, in which score 0 showed minimal diet quality, while score 100 was indicative of maximal diet quality. The DPI for each subject was calculated as: the total calorie of all phytochemical rich food components (including whole grains, fruits, vegetables, natural fruit and vegetable juices, soy products, tomato sauces, nuts, legumes, olive and olive oil) divided by total calorie intake.

### Assessment of genetic and biochemical parameters

Blood sampling carried out for all subjects after overnight fasting (8–12 h). Afterwards, blood samples were undergone either centrifugation or PCR analysis. The detailed description of PCR, TaqMan assay method for evaluation of ApoB *Ins/Del* and *EcoR1* SNPs have been published in our previous study^35^. The centrifugation was conducted for measurement of biochemical markers in the serum. Enzymatic method by commercially existing kits was used for determination of serum TC and triglyceride (TG) levels (Pars Azmoon, Iran), serum leptin (Bioassay Technology Co, China) and ghrelin (Bioassay Technology Co, Mediagnost, Germany) levels and serum IL-18, 8-iso-PGF2α and pentraxin-3 (PTX-3) levels (Shanghai Crystal Day Biotech Co., Ltd). Turbidimetry by a Roche Hitachi analyzer (Roche, Germany) was used for serum high density lipoprotein (HDL) and LDL levels determinations. Spectrophotometry procedure for serum TAC levels measurement was applied. Colorimetric procedure was used (Cayman Chemical Company, USA) for serum SOD levels measurements.

### Statistical analysis

The Kolmogorov–Smirnov (K-S) test was used to decide whether variables in population had a normal distribution. Transformations to square or logarithmic were used for abnormal data. There were three genotypes of ApoB *Ins/Del* SNP including *Ins/Ins*, *Ins/Del* and *Del/Del*. The genotypes of Apo B *EcoR1* SNP included *E* + *E* + , *E* + *E* − and *E* − *E* − . Independent t-test was applied for comparison of biochemical and anthropometric factors (BMI, WC, LDL, HDL, LDL/HDL, TC, TG, IL-18, TAC, PTX-3, SOD, iso-PGF2α, CRP, ghrelin and leptin) between Del allele carriers and subjects with Ins/Ins homozygous and between E − allele carriers and subjects with E + E + homozygous. HEI-2015, DQI-I and DPI scores were divided in to 3 equal intervals (tertile) for evaluating the adherence of subjects to indices. Analysis of covariance (ANCOVA) analysis test was carried out in order to compare the abovementioned study outcomes among groups of dietary indices.

Finally, interaction between ApoB Ins/Del SNP and dietary indices (DQI-I, HEI-2015 and DPI) as well as interaction between ApoB EcoR1 SNP and diet indices on the abovementioned variables were tested by using ANCOVA multivariate interaction models with and without adjusting for confounding factors (gender, age, smoking habits, and alcohol intake and physical activity). The results of interactions were presented as plots to visualize findings and help their illustrations.

In all stages of our research’s analysis, IBM SPSS (SPSS Inc., Chicago, IL, USA, version 21) was used, in which a *P*-value lower than 0.05 was assumed as significantly meaningful.

## Results

### Associations between DQI-I, HEI-2015 and DPI with biochemical and anthropometric parameters

Table 1 provides the findings of ANOVA test for comparing biochemical and anthropometric factors within the tertiles of dietary indices in recruited subjects. We observed significant differences in HDL (*P* = 0.01) and LDL/HDL (*P* = 0.04) levels between HEI-2015 tertiles. Our analysis did not display anymore significant or borderline significant difference in study’s variables within tertiles of DQI-I, HEI-2015 and DPI (*P* > 0.05).

**Table 1:** Assessment of the associations of dietary quality index-international, healthy eating index-2015 and dietary phytochemical index with cardio metabolic parameters.

Variable	Tertile 1	Tertile 2	Tertile 3	*P*-value*
**DQI-I**				
BMI (kg/m^2^)	29.70 ± 4.97	29.32 ± 4.60	28.92 ± 4.39	0.21
WC (cm)	92.42 ± 11.35	92.46 ± 9.92	91.39 ± 10.71	0.51
LDL (mg/dl)	108.27 ± 32.52	107.69 ± 32.54	110.59 ± 38.58	0.66
HDL (mg/dl)	53.08 ± 11.81	52.05 ± 11.09	53.22 ± 11.88	0.53
LDL/HDL	2.09 ± 0.65	3.00 ± 12.25	2.98 ± 12.64	0.53
TC (mg/dl)	195.18 ± 59.49	193.15 ± 58.22	191.84 ± 74.36	0.85
TG (mg/dl)	175.40 ± 95.57	171.22 ± 93.28	177.33 ± 107.00	0.81
Leptin (ng/ml)	25.75 ± 11.87	26.74 ± 17.23	24.44 ± 14.78	0.63
Ghrelin (ng/ml)	2.28 ± 1.27	2.23 ± 1.23	2.63 ± 1.53	0.15
CRP (mg/l)	1.81 ± 1.42	2.26 ± 1.52	2.50 ± 1.52	0.08
IL-18 (pg/ml)	255.16 ± 28.85	247.05 ± 29.00	246.09 ± 28.00	0.22
PTX-3 (ng/ml)	2.61 ± 0.39	2.66 ± 0.49	2.64 ± 0.51	0.87
TAC (g/dl)	2.53 ± 0.61	2.43 ± 0.56	2.43 ± 0.49	0.59
SOD (U/ml)	0.148 ± 0.04	0.144 ± 0.04	0.140 ± 0.04	0.70
8-iso-PGF2 α (pg/ml)	72.66 ± 5.46	71.96 ± 7.27	73.10 ± 5.97	0.65
**HEI-2015**				
BMI (kg/m^2^)	29.74 ± 4.65	29.36 ± 4.91	28.92 ± 4.45	0.18
WC (cm)	92.85 ± 10.93	92.29 ± 10.31	91.21 ± 10.86	0.27
LDL (mg/dl)	107.49 ± 33.95	107.72 ± 30.21	111.87 ± 39.62	0.33
HDL (mg/dl)	53.50 ± 11.57	51.02 ± 11.23	54.12 ± 12.04	**0.01**
LDL/HDL	2.05 ± 0.63	3.81 ± 17.04	2.11 ± 0.70	0.04
TC (mg/dl)	187.33 ± 59.08	197.67 ± 58.36	195.54 ± 74.07	0.20
TG (mg/dl)	173.97 ± 107.74	178.61 ± 100.57	172.00 ± 86.69	0.77
Leptin (ng/ml)	24.42 ± 13.56	27.87 ± 16.65	24.27 ± 13.67	0.25
Ghrelin (ng/ml)	2.33 ± 1.33	2.50 ± 1.31	2.37 ± 1.46	0.76
CRP (mg/l)	1.93 ± 1.50	2.53 ± 1.64	2.10 ± 1.34	0.13
IL-18 (pg/ml)	248.31 ± 29.04	250.67 ± 26.69	248.90 ± 30.68	0.91
PTX-3 (ng/ml)	2.62 ± 0.38	2.62 ± 0.47	2.66 ± 0.54	0.87
TAC (g/dl)	2.54 ± 0.56	2.50 ± 0.58	2.37 ± 0.51	0.27
SOD (U/ml)	0.156 ± 0.05	0.138 ± 0.04	0.140 ± 0.03	0.12
8-iso-PGF2 α (pg/ml)	72.22 ± 6.23	72.47 ± 6.36	73.06 ± 6.20	0.78
**DPI**				
BMI (kg/m^2^)	29.59 ± 4.96	29.26 ± 4.52	29.17 ± 4.56	0.62
WC (cm)	92.93 ± 10.71	91.76 ± 10.24	91.67 ± 11.17	0.40
LDL (mg/dl)	110.85 ± 34.55	105.47 ± 33.64	110.88 ± 36.18	0.17
HDL (mg/dl)	52.96 ± 11.96	53.58 ± 12.37	52.12 ± 10.64	0.42
LDL/HDL	2.14 ± 0.63	2.79 ± 11.51	3.01 ± 12.46	0.63
TC (mg/dl)	200.09 ± 80.18	192.95 ± 57.83	187.64 ± 51.64	0.13
TG (mg/dl)	189.41 ± 117.74	166.19 ± 78.56	169.38 ± 95.01	0.12
Leptin (ng/ml)	23.92 ± 13.42	26.79 ± 16.49	25.34 ± 13.73	0.52
Ghrelin (ng/ml)	2.39 ± 1.60	2.30 ± 1.23	2.49 ± 1.31	0.69
CRP (mg/l)	2.16 ± 1.42	2.20 ± 1.64	2.23 ± 1.47	0.96
IL-18 (pg/ml)	248.60 ± 28.77	249.67 ± 30.91	249.64 ± 26.83	0.97
PTX-3 (ng/ml)	2.62 ± 0.44	2.72 ± 0.48	2.57 ± 0.47	0.23
TAC (g/dl)	2.39 ± 0.50	2.46 ± 0.55	2.53 ± 0.59	0.45
SOD (U/ml)	0.146 ± 0.04	0.146 ± 0.05	0.140 ± 0.04	0.76
8-iso-PGF2 α (pg/ml)	72.37 ± 5.85	72.61 ± 6.20	72.79 ± 6.68	0.94

*P*-value*: ANOVA models. DQI-I: Dietary quality index-international, HEI-2015: Healthy eating index-U, DPI: Dietary phytochemical index, BMI: Body mass index, WC: waist circumference, LDL: Low density lipoprotein, HDL: High density lipoprotein, LDL/HDL: Low density lipoprotein / High density lipoprotein, TC: Total cholesterol, TG: Triglyceride, CRP: C-reactive protein, IL-18: Interleukin 18, PTX-3: Pentraxin-3, TAC: Total antioxidant capacity, SOD: super oxide dismutase and 8-iso-PGF2α: 8-iso-Prostaglandin F2α.

### Associations between ApoB Ins/Del and EcoR1 SNP and biochemical and anthropometric parameters

The comparison of risk factors of biochemical and anthropometric variables within genotypes of ApoB *Ins/Del* and *EcoR1* SNPs are presented in Table 2. A significant greater level of HDL was observed in *E* − allele carriers than subjects with *E* + *E* + homozygous (*P* = 0.001). No significant association was discovered between other study outcomes and investigated polymorphisms (*P* > 0.05).

**Table 2:** Assessment of the associations of ApoB *Ins/Del* and *EcoR1* polymorphisms with cardio metabolic parameters.

Variable	*Ins/Del* polymorphism			*EcoR1* polymorphism		
	*Ins/Del* and *Del/Del*	*Ins/Ins*	*P*-value	E −/E + and E −/E −	E +/E +	*P*-value
BMI (kg/m^2^)	29.18 ± 4.31	29.42 ± 4.86	0.40	30.28 ± 4.99	28.90 ± 4.48	0.16
WC (cm)	92.70 ± 10.90	91.46 ± 10.01	0.21	94.29 ± 11.95	91.33 ± 10.23	0.20
LDL (mg/dl)	113.01 ± 36.35	107.02 ± 33.91	0.71	108.57 ± 32.63	108.01 ± 36.28	0.20
HDL (mg/dl)	53.40 ± 11.78	52.63 ± 11.63	0.83	55.80 ± 15.73	52.72 ± 11.61	**0.001**
LDL/HDL	3.00 ± 12.41	2.47 ± 8.23	0.35	3.39 ± 15.84	2.46 ± 7.93	0.08
TC (mg/dl)	198.99 ± 70.10	190.73 ± 61.13	**0.001**	202.77 ± 82.58	200.92 ± 75.37	0.50
TG ( mg/dl)	175.82 ± 96.16	174.30 ± 99.82	0.41	179.82 ± 90.72	193.28 ± 114.77	0.18
Leptin (ng/ml)	27.04 ± 15.97	24.69 ± 13.97	0.46	26.12 ± 15.16	24.32 ± 14.00	0.93
Ghrelin (ng/ml)	2.27 ± 1.25	2.46 ± 1.42	0.17	2.39 ± 1.16	2.08 ± 1.15	0.94
CRP (mg/l)	2.20 ± 1.50	2.20 ± 1.52	0.86	2.46 ± 1.37	2.29 ± 1.53	0.16
IL-18 (pg/ml)	251.67 ± 28.22	248.07 ± 29.01	0.57	251. 48 ± 25.20	246.64 ± 32.27	0.13
Pentraxin 3 (ng/ml)	2.62 ± 0.46	2.64 ± 0.47	0.40	2.51 ± 0.44	2.66 ± 0.48	0.81
TAC (g/dl)	2.35 ± 0.52	2.52 ± 0.58	0.77	2.53 ± 0.60	2.61 ± 0.56	0.91
SOD (U/ml)	0.145 ± 0.04	0.143 ± 0.04	0.71	0.149 ± 0.04	0.142 ± 0.04	0.88
PGF2 α (pg/ml)	72.02 ± 5.95	72.92 ± 6.39	0.41	72.90 ± 6.17	72.35 ± 5.99	0.68

*P*-value*: Independent T-test models. BMI: Body mass index, WC: waist circumference, LDL: Low density lipoprotein, HDL: High density lipoprotein, LDL/HDL: Low density lipoprotein / High density lipoprotein, TC: Total cholesterol, TG: Triglyceride, CRP: C-reactive protein, IL-18: Interleukin 18, PTX-3: Pentraxin-3, TAC: Total antioxidant capacity, SOD: super oxide dismutase and 8-iso-PGF2α: 8-iso-Prostaglandin F2α.

Bold indicate *P*-values lower than 0.05.

### Interactions between Apo B Ins/Del and EcoR1 SNPs and dietary indices (DQI-I, HEI-2015 and DPI) on biochemical and anthropometric parameters

**Figure 1:**
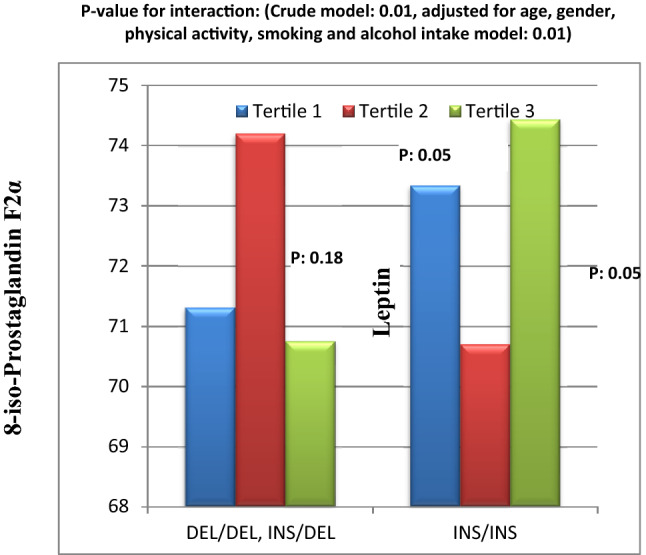
Interaction between Apo B INS/DEL SNP and DQI-I on serum prostaglandin F2α level.

**Figure 2:**
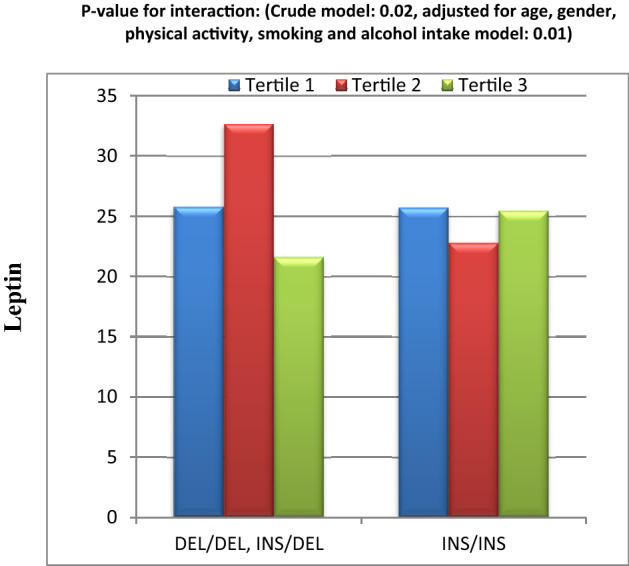
Interaction between Apo B INS/DEL SNP and DQI-I on serum leptin level.

**Figure 3:**
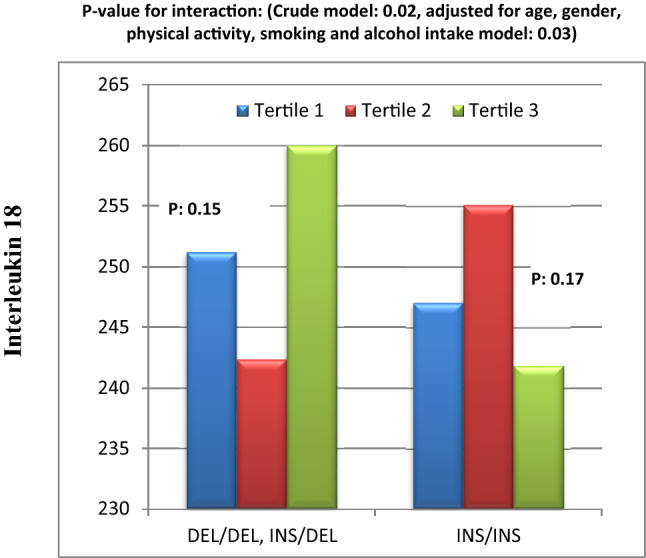
Interaction between Apo B INS/DEL SNP and HEI-2015 on serum interleukin 18 level.

**Figure 4:**
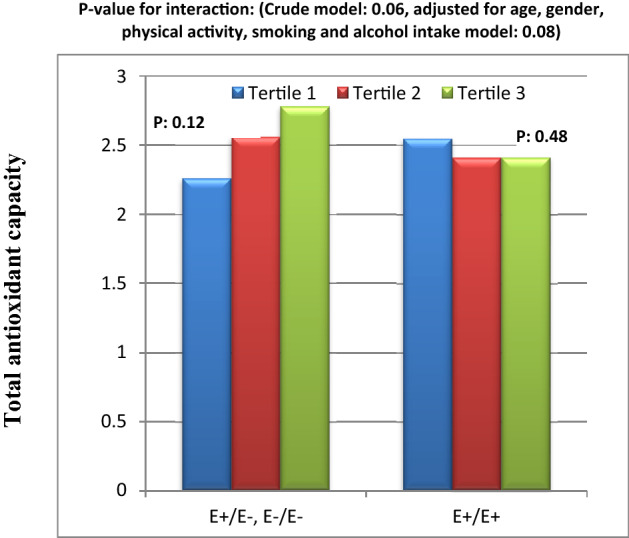
Interaction between Apo B EcoR1 SNP and DQI-I on serum total antioxidant capacity.

**Figure 5:**
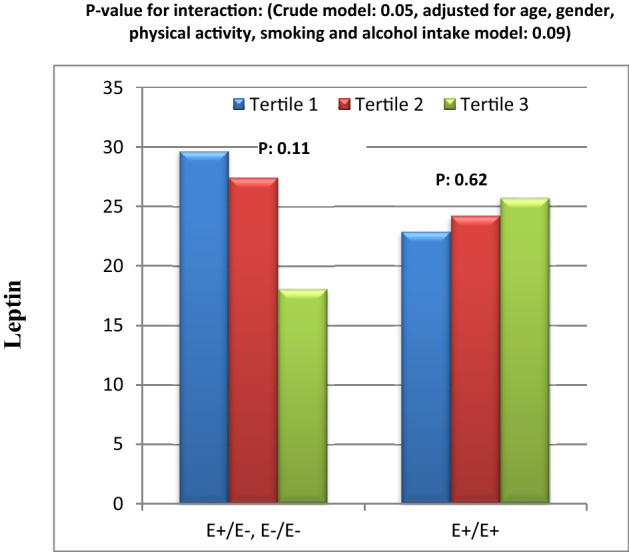
Interaction between Apo B EcoR1 SNP and HEI-2015 on serum leptin level.

**Figure 6:**
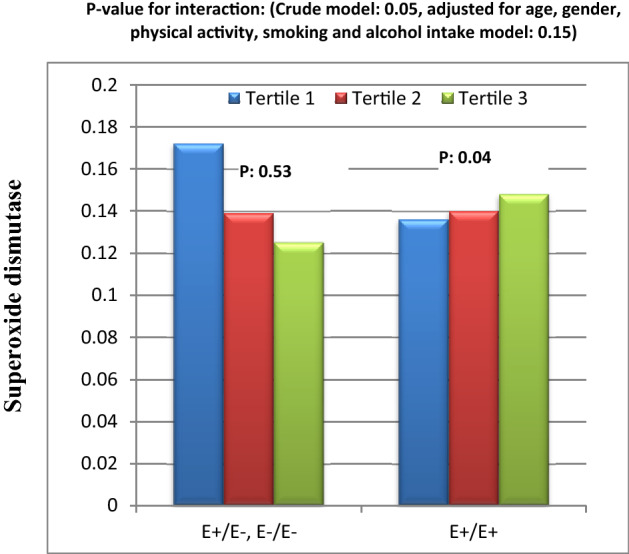
Interaction between Apo B EcoR1 SNP and HEI-2015 on serum superoxide dismutase level.

**Figure 7:**
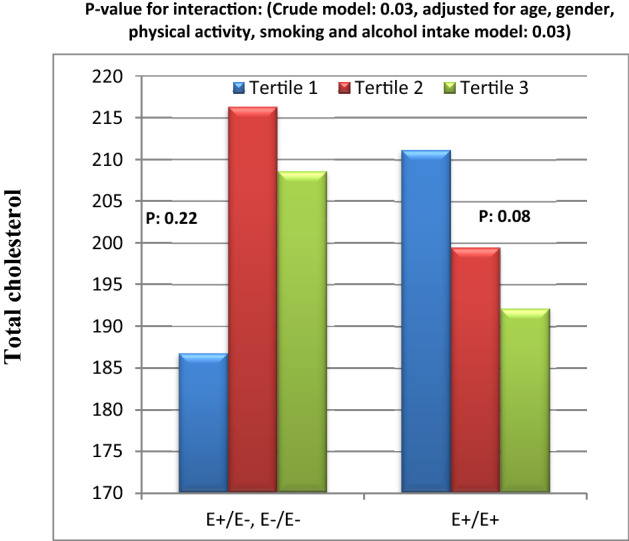
Interaction between Apo B EcoR1 SNP and DPI on serum total cholesterol level.

The results of the significant interactions are illustrated in Figs. 1, 2, 3, 4, 5, 6, and 7. Interaction between DQI-I score and Apo B Ins/Del SNP on serum 8-iso-PGF2α level was significant (Fig. 1). In fact, in people with Ins/Ins genotype, there was lower 8-iso-PGF2α concentration in tertile 2 of DQI-I score in comparison to tertile 1 and 3 of DQI-I score. However, in Del allele carriers, 8-iso-PGF2α level was significantly higher in tertile 2 of DQI-I score than tertile 1 and 3. This interaction was also significant after adjusting for confounding factors (gender, age, smoking habits, and alcohol intake and physical activity) (Fig. 1).

Moreover, there was significant interaction between DQI-I score and Apo B Ins/Del SNP on serum level of leptin was statically significant (Fig. 2). Based on Fig. 2, in Del allele carriers, leptin level was lower in tertile 2 than tertile 3 and 1. However, in Ins/Ins homozygous there was mildly lower leptin level in tertile 2 of DQI-I score compared to tertile 1 and 3. This interaction remained significant after adjusting for confounding factors (Fig. 2).

Findings of ANCOVA multivariate analysis also indicated a significant interaction between HEI-2015 score and Apo B Ins/Del SNP on serum IL-18 level in both crude and adjusted models (Fig. 3). In fact, in the interaction between HEI-2015 score and Apo B Ins/Del, level of IL-18 in patients with Ins/Ins genotype, was lower in tertile 1 and 3 than tertile 2 of HEI-2015 score. While in Del allele carriers, IL-18 concentration in tertile 1 and 3 of HEI-2015 was more than tertile 2 (Fig. 3).

Furthermore, the interaction test showed that in crude model, the interaction between ApoB EcoR1 SNP and DQI-I on serum TAC level was close to statistically significant (Fig. 4). As it is illustrated in Fig. 4, in E − allele carriers, there was increasing trend in TAC level from tertile 1 towards tertile 3 of HEI-2015. On the other hand, there was a mild higher TAC level in tertile 1 of HEI-2015 score than tertile 2 and 3 in people carrying E + E + genotype. This close to significant interaction disappeared after adjusting for confounding factors (Fig. 4).

In addition, in crude model, there were close to meaningful interactions between Apo B EcoR1 SNP and HEI-2015 score on serum leptin level in the participants which disappeared in adjusted model (Figs. 5). In Fig. 5, it can be observed that, there was a reducing trend in leptin level from tertile 1 towards tertile 3 of HEI-2015 in people carrying E − allele. However, this trend was milder and inversed in E + E + homozygous.

Furthermore, in crude model, there were borderline significant interaction between Apo B EcoR1 SNP and HEI-2015 score on SOD level (Fig. 6). In this interaction, while SOD concentration had a decreasing trend from tertile 1 towards tertile 3 of HEI-2015 in E − allele carriers, the level of SOD level increased from tertile 1 towards tertile 3 in participants with E + E + . This borderline significant interaction did not remain in adjusted model (Fig. 6).

Finally, we found a significant interaction between Apo B EcoR1 SNP and DPI score on serum TC level in both crude and adjusted models (Fig. 7). In fact, TC concentration had decreasing and increasing trends from tertile 1 towards tertile 3 of DPI score in E + E + homozygous and in E − allele carriers, respectively (Fig. 7).

ANCOVA multivariate interaction did not indicate any more significant or close to significant interactions between Apo B Ins/Del and EcoR1 SNPs and dietary quality indices (DQI-I, HEI-2015 and DPI) on study outcomes (Supplementary figures).

## Discussion

The present study indicated a significant interaction between ApoB *Ins/Del* SNP and HEI-2015 on serum IL-18 level. In subjects with *Ins/Ins* genotype, there were higher IL-18 levels in tertile 2 than those in tertiles 1 and 3 of HEI-2015. While the trend of the IL-18 level seemed to be inversed in *Del-*allele carries (i.e. subjects had higher IL-18 in the tertiles 1 and 3 of HEI-2015 than tertile 2). In fact, when HEI-2015 score was moderate*, Del* allele carriers and Ins/Ins homozygous had lower and higher IL-18 level, respectively. It has been reported that high blood IL-18 levels rise CVDs risk^36^. No precious mechanism has been proved yet, but some possible explanation could be proposed for the interaction of the Apo B Ins/Del polymorphism and HEI-2015 on IL-18 level. However there are possible explanations. For example, HEI-2015 considers high whole carbohydrate intakes as a part of healthy dietary pattern. People with T2DM often care to avoid eating refined carbohydrates and consume whole carbohydrates, instead. Consequently, participants with Ins/Ins genotype in tertile 2 of HEI-2015 consumed more whole carbohydrates and therefore higher total carbohydrates than Ins/Ins homozygous in tertile 1. In T2DM, higher total carbohydrate intake is associated with elevated inflammatory state^37^.

In conclusion, in Ins/Ins genotype carriers, higher total carbohydrate intake could have caused lower IL-18 level in tertile 2 compared to tertile 1. On the other hand, HEI-2015 focuses on main elements of a healthy diet such as limited consumption of SFA and added sugar as well as high intake of fruits, vegetables and nuts which have inflammation reducing properties^38,39^. Therefore, in Ins/Ins homozygous, in tertile 3 of HEI-2015, greater intakes of anti-inflammatory and antioxidant food components might have overcome the higher carbohydrates intakes and caused lower IL-18 levels in tertile 3 in comparison to tertile 2^40^.

As it was illustrated in Table 2, people with risk Del allele had more total cholesterol level than those with Ins/Ins genotype. There is currently evidence that Ins/Del SNP is related to deletion of 3 amino acids (Ala-Leu-Ala) from ApoB gene which alters the normal formation of recognition sit of ApoB for LDL recipient^41^. Del allele in this SNP may lead to dyslipidemia especially hypercholesterolemia^42^. It has been shown that people with hypercholesterolemia are more prone to inflammatory status reduction through diet or lifestyle interventions in comparison to people with healthy lipid profile^43^. Therefore, in Del-allele carriers, an increase in diet quality might have led to reduction in IL-18 level from tertile 1 towards tertile 2 of HEI-2015 score. On the other hand, persons with different kinds of hypercholesterolemia are more genetically predisposed to inflammatory status and premature CVDs events in response to high calorie intake than normal individuals^44^. Therefore, higher IL-18 in Del allele carriers in tertile 3 than tertile 2 and 1 of HEI-2015, might be due to the overcome of the adverse effect of greater calorie intakes on high healthy food components intake.

However, there are numerous factors that influence diet, which in turn can have an impact on various biomarkers. This calls for more investigations, and maybe alternative approaches, to determine the actual mechanism of these relationships.

According to our best knowledge to date, there is no research on the interaction between ApoB SNPs and dietary factors on IL-18 levels and other inflammatory cytokines. However, there are some interaction studies between lipid, SFA and cholesterol intakes and ApoB Ins/Del polymorphism on lipid profile that reported insignificant results on TC^20,45^. However, there was not significant interaction between *Ins/Del* and dietary indices on TC in our study. This inconsistency might be due to differences in study population and the interaction of other food parameters in dietary indices in this study with ApoB *Ins/Del* SNP. In our study, we also represented a significant interaction between DQI-I and *Ins/Del* variants on serum leptin concentration in both crude and adjusted models. It was observed that, serum leptin in individuals with *Ins/Ins* genotype was lower in tertile 3 and 1 of DQI-I than tertile 2. Although in those with *Del* allele, leptin level was not very different within DQI-I tertiles. A dietary pattern with high DQI-I and HEI-2015 scores is rich in MUFAs, PUFAs and fiber, poor in SFA and sugar and has a suitable proportion of total fat intake which can inhibit hypertriglyceridemia^46–48^. Low blood TG level can protect against leptin resistance via change in receptor signaling or metabolism of leptin^49^. Secondarily, we explored a borderline significant interaction between HEI-2015 and *EcoR1* SNP on leptin level which disappeared after controlling of confounding factors. There was a reducing trend in leptin concentration from tertile 1 toward tertile 3 of HEI-2015 in *E* − allele carriers, while there was only a mild increasing trend in subjects with *E* + *E* + homozygous. *EcoR1* SNP is related to the substitution of lysine for glutamic acid that alters the formation and tendency of recognition site for LDL receptor that might lead to hypercholesterolemia^50^. It appears that subjects with *Del* allele in *Ins/Del* SNP and *E* − allele in *EcoR1* SNP are more beneficially responsive to healthier diet than those with *Ins/Ins* and *E* + *E* + homozygous. Leptin is an adipokine which plays important roles in energy homeostasis and satiety. But hyperleptinemia and leptin resistance might be predictive factors of CVDs risk^51^. To our knowledge, only a study by Rafiee et al. assessed the interaction between dietary components and ApoB SNP on blood leptin levels in patients with T2DM. In their study, in Del allele carriers with T2DM, higher intake of MUFA, PUFA, SFA and protein and lower intake of carbohydrates were related to lower serum leptin concentration. However there was not any significant difference in subjects with Ins/Ins genotype^21^. Another finding of our study was the significant interaction between ApoB *EcoR1* polymorphism and DPI on serum TC concentrations in both crude and adjusted models. Based on our analysis, during tertiles of DPI in *E* + *E* + homozygous and *E* − allele carriers, TC levels had decreasing and increasing trends, respectively, indicating that phytochemical components may reduce cholesterol level in diabetic patients homozygous for *E* + allele through their anti-oxidative and anti-inflammatory characteristics^25,32^. Currently, there is no experimental or human study on the interaction between genetic profile and DPI on cardio metabolic parameters. However, some researched have demonstrated the reducing effect of DPI on inflammation, obesity and pre-diabetes^25,52^. Furthermore, the interaction between dietary patterns or components and ApoB SNPs on oxidative stress and anti-oxidative markers has not been investigated, yet. Present study indicated significant interaction between ApoB *Ins/Del* polymorphism and DQI-I on 8-iso-PGF2α in both crude and adjusted models. In fact, in *Ins/Ins* homozygous, moderate DQI-I score (tertile 2) was associated with benefits for oxidative stress shown by lower 8-iso-PGF2α levels compared to high (tertile 3) and low (tertile 1) DQI-I scores. On the other hand, there was increasing trend in 8-iso-PGF2α level from tertile 1 and 3 toward tertile 2 in *Del* allele carriers. Puchau B revealed a possible protective role of high DQI-I scores against oxidative stress in healthy subjects^53^. Therefore, in diabetic patients carrying *Del* allele, DQI-I appeared to have the strongest impact on reducing the serum 8-iso-PGF2α level, when it has the highest score due to high intake of antioxidant nutrients such as vitamin E, C, selenium, fiber^54,55^. While, it seems that only a moderate score of DQI-I is enough to reduce 8-iso-PGF2α level in subjects with Ins/Ins homozygous. Additionally, in subjects with Ins/Ins homozygous, high intakes of energy, refined grains and fruits in tertile 3 of DQI-I may have contributed to higher 8-iso-PGF2α compared to tertile 2. The change of tendency of ApoB to LDL recipients might explain variability in plasma 8-iso-PGF2α associations with DQI-I values between genotypes of *Ins/Del* polymorphism. In crude models, we also observed borderline significant interactions between ApoB *EcoR1* SNP and DQI-I on TAC level and between ApoB *EcoR1* SNP and HEI-2015 on SOD level. There was an elevating impact of high DQI-I scores in serum TAC in *E* − allele carriers. However, our results indicated that the response of SOD level as a main marker of antioxidant defense system to HEI-2015 was inversed and its concentrations decreased within HEI-2015 tertiles in *E* − allele carriers in crude model. But these borderline significant interactions on TAC and SOD disappeared after adjusting for confounding variables including BMI, age and smoking or alcohol uses. Findings of the relationship between HEI and oxidative stress are very limited. Two investigations have demonstrated that the HEI-2015 had a positive association with TAC and HEI-2010 had an insignificant association with SOD^56,57^. We further indicated that greater adherence to the HEI-2015 had favorable effects on serum HDL level and LDL/HDL ratio, respectively, which can be due to high intake of cardio protective micro-nutrients irrespective of differences in genotypes of ApoB SNPs^52^. In addition, serum HDL concentration was significantly higher in E − allele carriers in ApoB *EcoR1* SNP than *E* + *E* + homozygous. It should be noted that, high HDL level in *E* + *E* + homozygous in the current study can’t be interpreted as a cardio protective factor because some studies have indicated that genetic mutations that increase blood HDL level do not necessarily protects against CVDs^58^.

In this study, lack of causal interpretation due to cross-sectional design and measuring of ApoB serum level due to limited budget must be taken into consideration as limitations. However, our research is unique, because for the first time, we found that in T2DM patients carrying Del allele in ApoB *Ins/Del* SNP, moderate DQI-I values might reduce 8-iso-PGF2α and leptin as well as moderate HEI-2015 scores may be accompanied with reduction of IL-18. Additionally, we suspect that higher DPI in *E* + *E* + genotype of ApoB *EcoR1* is probably associated with lower serum TC in patients with T2DM. Further investigations are required to identify the exact underlying mechanisms of the observed interaction in this study.

## Supplementary Information

Supplementary Figures.

## Data Availability

Data sharing not applicable.
