# Supplementary material for: Dietary quality indices modify the effects of apolipoprotein B polymorphisms on biochemical and anthropometric factors in type 2 diabetes mellitus
Source: Sci Rep. 2021 Nov 17;11:22395. doi: 10.1038/s41598-021-01884-1 (PMC8599687; doi:10.1038/s41598-021-01884-1)
Supplement: Supplementary file 1 — Supplementary Figures. [file 41598_2021_1884_MOESM1_ESM.docx]

P-value for interaction: (Crude model: 0.53, adjusted for age, gender, physical activity, smoking and alcohol intake model: 0.33)

P-value for interaction: (Crude model: 0.20, adjusted for age, gender, physical activity, smoking and alcohol intake model: 0.18)

Body mass index

Waist circumference

Supplementary Figure 1: Interaction between Apo B INS/DEL SNP and DQI-I on body mass index

Supplementary Figure 2: Interaction between Apo B INS/DEL SNP and DQI-I on waist circumference

P-value for interaction: (Crude model: 0.97, adjusted for age, gender, physical activity, smoking and alcohol intake model: 0.88)

P-value for interaction: (Crude model: 0.73, adjusted for age, gender, physical activity, smoking and alcohol intake model: 0.76)

Low density lipoprotein

High density lipoprotein

Supplementary Figure 4: Interaction between Apo B INS/DEL SNP and DQI-I on high density lipoprotein

Supplementary Figure 3: Interaction between Apo B INS/DEL SNP and DQI-I on low density lipoprotein

P-value for interaction: (Crude model: 0.69, adjusted for age, gender, physical activity, smoking and alcohol intake model: 0.76)

P-value for interaction: (Crude model: 0.16, adjusted for age, gender, physical activity, smoking and alcohol intake model: 0.18)

Low density lipoprotein /High density lipoprotein

Total cholesterol

Supplementary Figure 5: Interaction between Apo B INS/DEL SNP and DQI-I on low density lipoprotein / high density lipoprotein

Supplementary Figure 6: Interaction between Apo B INS/DEL SNP and DQI-I on serum total cholesterol level

P-value for interaction: (Crude model: 0.48, adjusted for age, gender, physical activity, smoking and alcohol intake model: 0.38)

Triglyceride

Supplementary Figure 7: Interaction between Apo B INS/DEL SNP and DQI-I on serum triglyceride level

P-value for interaction: (Crude model: 0.73, adjusted for age, gender, physical activity, smoking and alcohol intake model: 0.74)

P-value for interaction: (Crude model: 0.32, adjusted for age, gender, physical activity, smoking and alcohol intake model: 0.25)

Ghrelin

C-reactive protein

Supplementary Figure 8: Interaction between Apo B INS/DEL SNP and DQI-I on serum ghrelin level

Supplementary Figure 9: Interaction between Apo B INS/DEL SNP and DQI-I on serum c-reactive protein level

P-value for interaction: (Crude model: 0.68, adjusted for age, gender, physical activity, smoking and alcohol intake model: 0.57)

P-value for interaction: (Crude model: 0.43, adjusted for age, gender, physical activity, smoking and alcohol intake model: 0.44)

Interleukin 18

Pentraxin 3

Supplementary Figure 10: Interaction between Apo B INS/DEL SNP and DQI-I on serum interleukin 18 level

Supplementary Figure 11: Interaction between Apo B INS/DEL SNP and DQI-I on serum pentraxin 3 level

P-value for interaction: (Crude model: 0.71, adjusted for age, gender, physical activity, smoking and alcohol intake model: 0.65)

P-value for interaction: (Crude model: 0.82, adjusted for age, gender, physical activity, smoking and alcohol intake model: 0.86)

Total antioxidant capacity

Superoxide dismutase

Supplementary Figure 12: Interaction between Apo B INS/DEL SNP and DQI-I on serum total antioxidant capacity

Supplementary Figure 13: Interaction between Apo B INS/DEL SNP and DQI-I on serum superoxide dismutase level

P-value for interaction: (Crude model: 0.30, adjusted for age, gender, physical activity, smoking and alcoh ol intake model: 0.42)

P-value for interaction: (Crude model: 0.45, adjusted for age, gender, physical activity, smoking and alcohol intake model: 0.49)

Body mass index

Waist circumference

Supplementary Figure 14: Interaction between Apo B INS/DEL SNP and HEI-B on body mass index

Supplementary Figure 15: Interaction between Apo B INS/DEL SNP and HEI-B on waist circumference

P-value for interaction: (Crude model: 0.35, adjusted for age, gender, physical activity, smoking and alcohol intake model: 0.38)

P-value for interaction: (Crude model: 0.61, adjusted for age, gender, physical activity, smoking and alcohol intake model: 0.56)

Low density lipoprotein

High density lipoprotein

Supplementary Figure 16: Interaction between Apo B INS/DEL SNP and HEI-B on low density lipoprotein

Supplementary Figure 17: Interaction between Apo B INS/DEL SNP and HEI-B on high density lipoprotein

P-value for interaction: (Crude model: 0.08, adjusted for age, gender, physical activity, smoking and alcohol intake model: 0.06)

P-value for interaction: (Crude model: 0.33, adjusted for age, gender, physical activity, smoking and alcohol intake model: 0.38)

Low density lipoprotein /High density lipoprotein

Total cholesterol

Supplementary Figure 18: Interaction between Apo B INS/DEL SNP and HEI-B on low density lipoprotein / high density lipoprotein

Supplementary Figure 19: Interaction between Apo B INS/DEL SNP and HEI-B on serum total cholesterol level

P-value for interaction: (Crude model: 0.54, adjusted for age, gender, physical activity, smoking and alcohol intake model: 0.54)

P-value for interaction: (Crude model: 0.23, adjusted for age, gender, physical activity, smoking and alcohol intake model: 0.21)

Triglyceride

Leptin

Supplementary Figure 20: Interaction between Apo B INS/DEL SNP and HEI-B on serum triglyceride level

Supplementary Figure 21: Interaction between Apo B INS/DEL SNP and HEI-B on serum leptin level

P-value for interaction: (Crude model: 0.88, adjusted for age, gender, physical activity, smoking and alcohol intake model: 0.81)

P-value for interaction: (Crude model: 0.80, adjusted for age, gender, physical activity, smoking and alcohol intake model: 0.73)

Ghrelin

C-reactive protein

Supplementary Figure 22: Interaction between Apo B INS/DEL SNP and HEI-B on serum ghrelin level

Supplementary Figure 23: Interaction between Apo B INS/DEL SNP and HEI-B on serum c-reactive protein level

P-value for interaction: (Crude model: 0.90, adjusted for age, gender, physical activity, smoking and alcohol intake model: 0.89)

P-value for interaction: (Crude model: 0.10, adjusted for age, gender, physical activity, smoking and alcohol intake model: 0.08)

Interleukin 18

Pentraxin 3

Supplementary Figure 24: Interaction between Apo B INS/DEL SNP and HEI-B on serum interleukin 18 level

Supplementary Figure 25: Interaction between Apo B INS/DEL SNP and HEI-B on serum pentraxin 3 level

P-value for interaction: (Crude model: 0.55, adjusted for age, gender, physical activity, smoking and alcohol intake model: 0.30)

P-value for interaction: (Crude model: 0.30, adjusted for age, gender, physical activity, smoking and alcohol intake model: 0.16)

Total antioxidant capacity

Superoxide dismutase

Supplementary Figure 26: Interaction between Apo B INS/DEL SNP and HEI-B on serum total antioxidant capacity level

Supplementary Figure 27: Interaction between Apo B INS/DEL SNP and HEI-B on serum superoxide dismutase level

P-value for interaction: (Crude model: 0.23, adjusted for age, gender, physical activity, smoking and alcohol intake model: 0.20)

Prostaglandin F2α

Supplementary Figure 28: Interaction between Apo B INS/DEL SNP and HEI-B on serum prostaglandin F2α level

P-value for interaction: (Crude model: 0.54, adjusted for age, gender, physical activity, smoking and alcohol intake model: 0.86)

P-value for interaction: (Crude model: 0.67, adjusted for age, gender, physical activity, smoking and alcohol intake model: 0.53)

Body mass index

Waist circumference

Supplementary Figure 29: Interaction between Apo B INS/DEL SNP and HEI-U on body mass index

Supplementary Figure 30: Interaction between Apo B INS/DEL SNP and HEI-U on waist circumference

P-value for interaction: (Crude model: 0.63, adjusted for age, gender, physical activity, smoking and alcohol intake model: 0.33)

P-value for interaction: (Crude model: 0.24, adjusted for age, gender, physical activity, smoking and alcohol intake model: 0.27)

Low density lipoprotein

High density lipoprotein

Supplementary Figure 31: Interaction between Apo B INS/DEL SNP and HEI-U on low density lipoprotein

Supplementary Figure 32: Interaction between Apo B INS/DEL SNP and HEI-U on high density lipoprotein

P-value for interaction: (Crude model: 0.78, adjusted for age, gender, physical activity, smoking and alcohol intake model: 0.83)

P-value for interaction: (Crude model: 0.77, adjusted for age, gender, physical activity, smoking and alcohol intake model: 0.73)

Low density lipoprotein /High density lipoprotein

Total cholesterol

Supplementary Figure 33: Interaction between Apo B INS/DEL SNP and HEI-U on low density lipoprotein / high density lipoprotein

Supplementary Figure 34: Interaction between Apo B INS/DEL SNP and HEI-U on serum total cholesterol level

P-value for interaction: (Crude model: 0.29, adjusted for age, gender, physical activity, smoking and alcohol intake model: 0.59)

P-value for interaction: (Crude model: 0.35, adjusted for age, gender, physical activity, smoking and alcohol intake model: 0.28)

Triglyceride

Leptin

Supplementary Figure 35: Interaction between Apo B INS/DEL SNP and HEI-U on serum triglyceride level

Supplementary Figure 36: Interaction between Apo B INS/DEL SNP and HEI-U on serum leptin level

P-value for interaction: (Crude model: 0.80, adjusted for age, gender, physical activity, smoking and alcohol intake model: 0.73)

P-value for interaction: (Crude model: 0.58, adjusted for age, gender, physical activity, smoking and alcohol intake model: 0.62)

Ghrelin

C-reactive protein

Supplementary Figure 37: Interaction between Apo B INS/DEL SNP and HEI-U on serum ghrelin level

Supplementary Figure 38: Interaction between Apo B INS/DEL SNP and HEI-U on serum c-reactive protein level

P-value for interaction: (Crude model: 0.41, adjusted for age, gender, physical activity, smoking and alcohol intake model: 0.45)

Pentraxin 3

Supplementary Figure 39: Interaction between Apo B INS/DEL SNP and HEI-U on serum pentraxin 3 level

P-value for interaction: (Crude model: 0.84, adjusted for age, gender, physical activity, smoking and alcohol intake model: 0.83)

P-value for interaction: (Crude model: 0.72, adjusted for age, gender, physical activity, smoking and alcohol intake model: 0.67)

Total antioxidant capacity level

Superoxide dismutase

Supplementary Figure 41: Interaction between Apo B INS/DEL SNP and HEI-U on serum superoxide dismutase level

Supplementary Figure 40: Interaction between Apo B INS/DEL SNP and HEI-U on serum total antioxidant capacity level

P-value for interaction: (Crude model: 0.77, adjusted for age, gender, physical activity, smoking and alcohol intake model: 0.86)

Prostaglandin F2α

Supplementary Figure 42: Interaction between Apo B INS/DEL SNP and HEI-U on serum prostaglandin F2α level

P-value for interaction: (Crude model: 0.53, adjusted for age, gender, physical activity, smoking and alcohol intake model: 0.61)

P-value for interaction: (Crude model: 0.20, adjusted for age, gender, physical activity, smoking and alcohol intake model: 0.87)

Body mass index

Waist circumference

Supplementary Figure 43: Interaction between Apo B INS/DEL SNP and DPI on body mass index

Supplementary Figure 44: Interaction between Apo B INS/DEL SNP and DPI on waist circumference

P-value for interaction: (Crude model: 0.97, adjusted for age, gender, physical activity, smoking and alcohol intake model: 0.64)

P-value for interaction: (Crude model: 0.73, adjusted for age, gender, physical activity, smoking and alcohol intake model: 0.14)

Low density lipoprotein

High density lipoprotein

Figure 45: Interaction between Apo B INS/DEL SNP and DPI on low density lipoprotein

Figure 46: Interaction between Apo B INS/DEL SNP and DPI on high density lipoprotein

P-value for interaction: (Crude model: 0.08, adjusted for age, gender, physical activity, smoking and alcohol intake model: 0.11)

P-value for interaction: (Crude model: 0.42, adjusted for age, gender, physical activity, smoking and alcohol intake model: 0.35)

Low density lipoprotein /High density lipoprotein

Total cholesterol

Supplementary Figure 47: Interaction between Apo B INS/DEL SNP and DPI on low density lipoprotein / high density lipoprotein

Supplementary Figure 48: Interaction between Apo B INS/DEL SNP and DPI on serum total cholesterol level

P-value for interaction: (Crude model: 0.23, adjusted for age, gender, physical activity, smoking and alcohol intake model: 0.14)

P-value for interaction: (Crude model: 0.38, adjusted for age, gender, physical activity, smoking and alcohol intake model: 0.31)

Triglyceride

Leptin

Supplementary Figure 49: Interaction between Apo B INS/DEL SNP and DPI on serum triglyceride level

Supplementary Figure 50: Interaction between Apo B INS/DEL SNP and DPI on serum leptin level

P-value for interaction: (Crude model: 0.09, adjusted for age, gender, physical activity, smoking and alcohol intake model: 0.09)

P-value for interaction: (Crude model: 0.46, adjusted for age, gender, physical activity, smoking and alcohol intake model: 0.65)

P: 0.

C-reactive protein

Ghrelin

Supplementary Figure 51: Interaction between Apo B INS/DEL SNP and DPI on serum ghrelin level

Supplementary Figure 52: Interaction between Apo B INS/DEL SNP and DPI on serum c-reactive protein level

P-value for interaction: (Crude model: 0.12, adjusted for age, gender, physical activity, smoking and alcohol intake model: 0.09)

P-value for interaction: (Crude model: 0.67, adjusted for age, gender, physical activity, smoking and alcohol intake model: 0.57)

Interleukin 18

Pentraxin 3

Supplementary Figure 53: Interaction between Apo B INS/DEL SNP and DPI on serum interleukin 18 level

Supplementary Figure 54: Interaction between Apo B INS/DEL SNP and DPI on serum pentraxin 3 level

P-value for interaction: (Crude model: 0.60, adjusted for age, gender, physical activity, smoking and alcohol intake model: 0.68)

P-value for interaction: (Crude model: 0.64, adjusted for age, gender, physical activity, smoking and alcohol intake model: 0.56)

Total antioxidant capacity

Superoxide dismutase

Supplementary Figure 55: Interaction between Apo B INS/DEL SNP and DPI on serum total antioxidant capacity level

Supplementary Figure 56: Interaction between Apo B INS/DEL SNP and DPI on serum superoxide dismutase level

P-value for interaction: (Crude model: 0.14, adjusted for age, gender, physical activity, smoking and alcohol intake model: 0.16)

Prostaglandin F2α

Supplementary Figure 57: Interaction between Apo B INS/DEL SNP and DPI on serum prostaglandin F2α level

P-value for interaction: (Crude model: 0.84, adjusted for age, gender, physical activity, smoking and alcohol intake model: 0.80)

P-value for interaction: (Crude model: 0.30, adjusted for age, gender, physical activity, smoking and alcohol intake model: 0.28)

Body mass index

Waist circumference

Supplementary Figure 59: Interaction between Apo B EcoR1 SNP and DQI-I on waist circumference

Supplementary Figure 58: Interaction between Apo B EcoR1 SNP and DQI-I on body mass index

P-value for interaction: (Crude model: 0.62, adjusted for age, gender, physical activity, smoking and alcohol intake model: 0.57)

P-value for interaction: (Crude model: 0.76, adjusted for age, gender, physical activity, smoking and alcohol intake model: 0.79)

Low density lipoprotein

High density lipoprotein

Supplementary Figure 60: Interaction between Apo B EcoR1 SNP and DQI-I on low density lipoprotein

Supplementary Figure 61: Interaction between Apo B EcoR1 SNP and DQI-I on high density lipoprotein

P-value for interaction: (Crude model: 0.69, adjusted for age, gender, physical activity, smoking and alcohol intake model: 0.71)

P-value for interaction: (Crude model: 0.33, adjusted for age, gender, physical activity, smoking and alcohol intake model: 0.28)

Low density lipoprotein /High density lipoprotein

Total cholesterol

Supplementary Figure 62: Interaction between Apo B EcoR1 SNP and DQI-I on low density lipoprotein / high density lipoprotein

Supplementary Figure 63: Interaction between Apo B EcoR1 SNP and DQI-I on serum total cholesterol level

P-value for interaction: (Crude model: 0.94, adjusted for age, gender, physical activity, smoking and alcohol intake model: 0.95)

P-value for interaction: (Crude model: 0.73, adjusted for age, gender, physical activity, smoking and alcohol intake model: 0.76)

Leptin

Triglyceride

Supplementary Figure 65: Interaction between Apo B EcoR1 SNP and DQI-I on serum leptin level

Supplementary Figure 64: Interaction between Apo B EcoR1 SNP and DQI-I on serum triglyceride level

P-value for interaction: (Crude model: 0.45, adjusted for age, gender, physical activity, smoking and alcohol intake model: 0.28)

P-value for interaction: (Crude model: 0.75, adjusted for age, gender, physical activity, smoking and alcohol intake model: 0.93)

Ghrelin

C-reactive protein

Supplementary Figure 66: Interaction between Apo B EcoR1 SNP and DQI-I on serum ghrelin level

Supplementary Figure 67: Interaction between Apo B EcoR1 SNP and DQI-I on serum c-reactive protein level

P-value for interaction: (Crude model: 0.32, adjusted for age, gender, physical activity, smoking and alcohol intake model: 0.25)

P-value for interaction: (Crude model: 0.22, adjusted for age, gender, physical activity, smoking and alcohol intake model: 0.21)

Interleukin 18

Pentraxin 3

Supplementary Figure 69: Interaction between Apo B EcoR1 SNP and DQI-I on serum pentraxin 3 level

Supplementary Figure 68: Interaction between Apo B EcoR1 SNP and DQI-I on serum interleukin 18 level

P-value for interaction: (Crude model: 0.24, adjusted for age, gender, physical activity, smoking and alcohol intake model: 0.26)

P-value for interaction: (Crude model: 0.58, adjusted for age, gender, physical activity, smoking and alcohol intake model: 0.71)

Prostaglandin F2α

Superoxide dismutase

Supplementary Figure 71: Interaction between Apo B EcoR1 SNP and DQI-I on serum prostaglandin F2α level

Supplementary Figure 70: Interaction between Apo B EcoR1 SNP and DQI-I on serum superoxide dismutase level

P-value for interaction: (Crude model: 0.35, adjusted for age, gender, physical activity, smoking and alcohol intake model: 0.52)

P-value for interaction: (Crude model: 0.89, adjusted for age, gender, physical activity, smoking and alcohol intake model: 0.79)

Body mass index

Waist circumference

Supplementary Figure 72: Interaction between Apo B EcoR1 SNP and HEI-B on body mass index

Supplementary Figure 73: Interaction between Apo B EcoR1 SNP and HEI-B on waist circumference

P-value for interaction: (Crude model: 0.69, adjusted for age, gender, physical activity, smoking and alcohol intake model: 0.78)

P-value for interaction: (Crude model: 0.81, adjusted for age, gender, physical activity, smoking and alcohol intake model: 0.88)

Low density lipoprotein

High density lipoprotein

Supplementary Figure 74: Interaction between Apo B EcoR1 SNP and HEI-B on low density lipoprotein

Supplementary Figure 75: Interaction between Apo B EcoR1 SNP and HEI-B on high density lipoprotein

P-value for interaction: (Crude model: 0.26, adjusted for age, gender, physical activity, smoking and alcohol intake model: 0.21)

P-value for interaction: (Crude model: 0.15, adjusted for age, gender, physical activity, smoking and alcohol intake model: 0.18)

Low density lipoprotein /High density lipoprotein

Total cholesterol

Supplementary Figure 76: Interaction between Apo B EcoR1 SNP and HEI-B on low density lipoprotein / high density lipoprotein

Supplementary Figure 77: Interaction between Apo B EcoR1 SNP and HEI-B on serum total cholesterol level

P-value for interaction: (Crude model: 0.42, adjusted for age, gender, physical activity, smoking and alcohol intake model: 0.56)

P-value for interaction: (Crude model: 0.84, adjusted for age, gender, physical activity, smoking and alcohol intake model: 0.83)

Triglyceride

Leptin

Supplementary Figure 78: Interaction between Apo B EcoR1 SNP and HEI-B on serum triglyceride level

Supplementary Figure 79: Interaction between Apo B EcoR1 SNP and HEI-B on serum leptin level

P-value for interaction: (Crude model: 0.57, adjusted for age, gender, physical activity, smoking and alcohol intake model: 0.57)

P-value for interaction: (Crude model: 0.68, adjusted for age, gender, physical activity, smoking and alcohol intake model: 0.62)

Ghrelin

C-reactive protein

Supplementary Figure 80: Interaction between Apo B EcoR1 SNP and HEI-B on serum ghrelin level

Supplementary Figure 81: Interaction between Apo B EcoR1 SNP and HEI-B on serum c-reactive protein level

P-value for interaction: (Crude model: 0.95, adjusted for age, gender, physical activity, smoking and alcohol intake model: 0.95)

P-value for interaction: (Crude model: 0.41, adjusted for age, gender, physical activity, smoking and alcohol intake model: 0.42)

Interleukin 18

Pentraxin 3

Supplementary Figure 82: Interaction between Apo B EcoR1 SNP and HEI-B on serum interleukin 18 level

Supplementary Figure 83: Interaction between Apo B EcoR1 SNP and HEI-B on serum pentraxin 3 level

P-value for interaction: (Crude model: 0.47, adjusted for age, gender, physical activity, smoking and alcohol intake model: 0.37)

P-value for interaction: (Crude model: 0.16, adjusted for age, gender, physical activity, smoking and alcohol intake model: 0.16)

Total antioxidant capacity

Superoxide dismutase

Supplementary Figure 84: Interaction between Apo B EcoR1 SNP and HEI-B on serum total antioxidant capacity level

Supplementary Figure 85: Interaction between Apo B EcoR1 SNP and HEI-B on serum superoxide dismutase level

P-value for interaction: (Crude model: 0.29, adjusted for age, gender, physical activity, smoking and alcohol intake model: 0.27)

Prostaglandin F2α

Supplementary Figure 86: Interaction between Apo B EcoR1 SNP and HEI-B on serum prostaglandin F2α level

P-value for interaction: (Crude model: 0.22, adjusted for age, gender, physical activity, smoking and alcohol intake model: 0.41)

P-value for interaction: (Crude model: 0.41, adjusted for age, gender, physical activity, smoking and alcohol intake model: 0.31)

Body mass index

Waist circumference

Supplementary Figure 87: Interaction between Apo B EcoR1 SNP and HEI-U on body mass index

Supplementary Figure 88: Interaction between Apo B EcoR1 SNP and HEI-U on waist circumference

P-value for interaction: (Crude model: 0.08, adjusted for age, gender, physical activity, smoking and alcohol intake model: 0.29)

P-value for interaction: (Crude model: 0.96, adjusted for age, gender, physical activity, smoking and alcohol intake model: 0.95)

Low density lipoprotein

High density lipoprotein

Supplementary Figure 89: Interaction between Apo B EcoR1 SNP and HEI-U on low density lipoprotein

Supplementary Figure 90: Interaction between Apo B EcoR1 SNP and HEI-U on high density lipoprotein

P-value for interaction: (Crude model: 0.11, adjusted for age, gender, physical activity, smoking and alcohol intake model: 0.14)

P-value for interaction: (Crude model: 0.21, adjusted for age, gender, physical activity, smoking and alcohol intake model: 0.28)

Low density lipoprotein /High density lipoprotein

Total cholesterol

Supplementary Figure 91: Interaction between Apo B EcoR1 SNP and HEI-U on low density lipoprotein / high density lipoprotein

Supplementary Figure 92: Interaction between Apo B EcoR1 SNP and HEI-U on serum total cholesterol level

P-value for interaction: (Crude model: 0.98, adjusted for age, gender, physical activity, smoking and alcohol intake model: 0.99)

Triglyceride

Supplementary Figure 93: Interaction between Apo B EcoR1 SNP and HEI-U on serum triglyceride level

P-value for interaction: (Crude model: 0.97, adjusted for age, gender, physical activity, smoking and alcohol intake model: 0.89)

P-value for interaction: (Crude model: 0.74, adjusted for age, gender, physical activity, smoking and alcohol intake model: 0.82)

P:

Ghrelin

C-reactive protein

Supplementary Figure 94: Interaction between Apo B EcoR1 SNP and HEI-U on serum ghrelin level

Supplementary Figure 95: Interaction between Apo B EcoR1 SNP and HEI-U on serum C-reactive protein level

P-value for interaction: (Crude model: 0.50, adjusted for age, gender, physical activity, smoking and alcohol intake model: 0.45)

P-value for interaction: (Crude model: 0.89, adjusted for age, gender, physical activity, smoking and alcohol intake model: 0.89)

Interleukin 18

Pentraxin 3

Supplementary Figure 97: Interaction between Apo B EcoR1 SNP and HEI-U on serum pentraxin 3 level

Supplementary Figure 96: Interaction between Apo B EcoR1 SNP and HEI-U on serum interleukin 18 level

P-value for interaction: (Crude model: 0.56, adjusted for age, gender, physical activity, smoking and alcohol intake model: 0.47)

P-value for interaction: (Crude model: 0.68, adjusted for age, gender, physical activity, smoking and alcohol intake model: 0.86)

Total antioxidant capacity level

Prostaglandin F2α

Supplementary Figure 99: Interaction between Apo B EcoR1 SNP and HEI-U on serum prostaglandin F2α level

Supplementary Figure 98: Interaction between Apo B EcoR1 SNP and HEI-U on serum total antioxidant capacity level

P-value for interaction: (Crude model: 0.51, adjusted for age, gender, physical activity, smoking and alcohol intake model: 0.47)

P-value for interaction: (Crude model: 0.54, adjusted for age, gender, physical activity, smoking and alcohol intake model: 0.36)

Body mass index

Waist circumference

Supplementary Figure 100: Interaction between Apo B EcoR1 SNP and DPI on body mass index

Supplementary Figure 101: Interaction between Apo B EcoR1 SNP and DPI on waist circumference

P-value for interaction: (Crude model: 0.93, adjusted for age, gender, physical activity, smoking and alcohol intake model: 0.97)

P-value for interaction: (Crude model: 0.70, adjusted for age, gender, physical activity, smoking and alcohol intake model: 0.68)

Low density lipoprotein

High density lipoprotein

Supplementary Figure 102: Interaction between Apo B EcoR1 SNP and DPI on low density lipoprotein

Supplementary Figure 103: Interaction between Apo B EcoR1 SNP and DPI on high density lipoprotein

P-value for interaction: (Crude model: 0.25, adjusted for age, gender, physical activity, smoking and alcohol intake model: 0.23)

Low density lipoprotein /High density lipoprotein

Supplementary Figure 104: Interaction between Apo B EcoR1 SNP and DPI on low density lipoprotein / high density lipoprotein

P-value for interaction: (Crude model: 0.48, adjusted for age, gender, physical activity, smoking and alcohol intake model: 0.31)

P-value for interaction: (Crude model: 0.29, adjusted for age, gender, physical activity, smoking and alcohol intake model: 0.31)

Triglyceride

Leptin

Supplementary Figure 105: Interaction between Apo B EcoR1 SNP and DPI on serum triglyceride level

Supplementary Figure 106: Interaction between Apo B EcoR1 SNP and DPI on serum leptin level

P-value for interaction: (Crude model: 0.43, adjusted for age, gender, physical activity, smoking and alcohol intake model: 0.36)

P-value for interaction: (Crude model: 0.61, adjusted for age, gender, physical activity, smoking and alcohol intake model: 0.64)

Ghrelin

C-reactive protein

Supplementary Figure 107: Interaction between Apo B EcoR1 SNP and DPI on serum ghrelin level

Supplementary Figure 108: Interaction between Apo B EcoR1 SNP and DPI on serum c-reactive protein level

P-value for interaction: (Crude model: 0.74, adjusted for age, gender, physical activity, smoking and alcohol intake model: 0.79)

P-value for interaction: (Crude model: 0.34, adjusted for age, gender, physical activity, smoking and alcohol intake model: 0.42)

Interleukin 18

Pentraxin 3

Supplementary Figure 109: Interaction between Apo B EcoR1 SNP and DPI on serum interleukin 18 level

Supplementary Figure 110: Interaction between Apo B EcoR1 SNP and DPI on serum pentraxin 3 level

P-value for interaction: (Crude model: 0.38, adjusted for age, gender, physical activity, smoking and alcohol intake model: 0.53)

P-value for interaction: (Crude model: 0.93, adjusted for age, gender, physical activity, smoking and alcohol intake model: 0.86)

Total antioxidant capacity

Superoxide dismutase

Supplementary Figure 111: Interaction between Apo B EcoR1 SNP and DPI on serum total antioxidant capacity level

Supplementary Figure 112: Interaction between Apo B EcoR1 SNP and DPI on serum superoxide dismutase level

P-value for interaction: (Crude model: 0.15, adjusted for age, gender, physical activity, smoking and alcohol intake model: 0.24)

Prostaglandin F2α

Supplementary Figure 113: Interaction between Apo B EcoR1 SNP and DPI on serum prostaglandin F2α level
